# Ultrasound-Based Severity Stratification in Carpal Tunnel Syndrome: Median Nerve Cross-Sectional Area for Distinguishing Severe from Moderate Disease

**DOI:** 10.3390/diagnostics16152458

**Published:** 2026-08-04

**Authors:** Işıl Peker, Abir Alaamel, Nilgün Cengiz

**Affiliations:** 1 Department of Neurology, Faculty of Medicine, Trakya University, 22030 Edirne, Türkiye; 2 Department of Neurology, Edirne Sultan 1. Murat State Hospital, 22030 Edirne, Türkiye

**Keywords:** carpal tunnel syndrome, median nerve, ultrasound, cross-sectional area, nerve conduction studies, needle electromyography, disease severity

## Abstract

**Background:** High-resolution ultrasound is increasingly used as a complementary tool in carpal tunnel syndrome (CTS), but the value of median nerve cross-sectional area (CSA) for severity stratification remains uncertain. This study evaluated whether median nerve CSA measured at the pisiform level can distinguish severe from moderate CTS classified according to the Bland grading scale. **Methods:** This prospective cross-sectional study included 72 participants: moderate CTS (*n* = 34), severe CTS (*n* = 24), and healthy controls (*n* = 14). Nerve conduction studies, abductor pollicis brevis needle electromyography, and ultrasound examinations were performed on the same day. CSA was measured three times by a blinded examiner, and the mean value was used for analysis. Correlation, age-adjusted linear regression, and receiver operating characteristic curve analyses were performed. **Results:** Median nerve CSA increased progressively from controls to moderate and severe CTS groups (8.0 ± 1.1, 16.5 ± 4.6, and 19.8 ± 3.4 mm^2^, respectively; *p* < 0.001), with significant pairwise differences. Intraobserver and interobserver reliability were excellent (ICC = 0.95 and 0.91, respectively). CSA correlated positively with distal motor and sensory latencies and inversely with compound muscle action potential and sensory nerve action potential amplitudes. CSA also showed a weak positive association with neurogenic motor unit action potentials. In age-adjusted regression analysis, distal motor latency remained independently associated with CSA (B = 0.873, *p* = 0.002). A CSA cut-off of 18.5 mm^2^ distinguished severe from moderate CTS with an area under the curve (AUC) of 0.728, sensitivity of 66.67%, and specificity of 79.41%. **Conclusions:** Median nerve CSA measured at the pisiform level may provide useful adjunctive structural information for CTS severity assessment and may show moderate discriminatory performance for differentiating severe from moderate CTS. CSA should be interpreted in conjunction with clinical and electrodiagnostic findings rather than as a stand-alone severity marker.

## 1. Introduction

Carpal tunnel syndrome (CTS) is the most common entrapment neuropathy and arises from compression of the median nerve at the wrist [1]. Its clinical presentation spans a broad spectrum, ranging from intermittent paraesthesia to persistent sensory impairment, thenar weakness, and ultimately thenar atrophy in advanced cases [1]. Beyond diagnostic confirmation, accurate assessment of disease severity is clinically important, as increasing severity may indicate greater median nerve dysfunction, possible axonal involvement, and implications for treatment planning [2].

Nerve conduction studies (NCS) provide functional information regarding median nerve impairment and remain central to electrodiagnostic severity grading in CTS [3]. The Bland grading scale offers a structured and widely used electrodiagnostic classification system for CTS severity in both clinical practice and research [4]. In addition, needle electromyography (EMG) of the abductor pollicis brevis (APB) muscle may provide complementary information, particularly for detecting axonal loss in more advanced disease [5].

Complementing the functional assessment provided by electrodiagnostic studies, high-resolution ultrasound (US) enables direct visualisation of the median nerve and provides morphological information on nerve enlargement and potential local compressive factors [6,7]. Among sonographic parameters, the cross-sectional area (CSA) of the median nerve at the carpal tunnel inlet is the most widely used, reproducible, and clinically accessible measurement [8,9]. Nevertheless, reported CSA thresholds and diagnostic accuracy estimates vary considerably across studies [9,10]. In addition, CSA measurement may be influenced by operator- and acquisition-related factors. Recent evidence indicates that evaluator experience, transducer frequency, and dynamic versus static image interpretation affect the differentiation of intraneural fascicles [11]. These findings underscore the importance of technical expertise and careful delineation of nerve boundaries when measuring CSA.

Although enlargement of the median nerve CSA is a well-established sonographic feature of CTS and ultrasound has shown acceptable diagnostic accuracy for CTS diagnosis [9], diagnostic confirmation and severity stratification represent distinct clinical questions. In particular, the ability of CSA to discriminate moderate from severe CTS is clinically relevant, as this distinction may reflect more advanced nerve dysfunction, possible axonal involvement, and potential differences in clinical management. Previous studies examining the association between median nerve CSA and electrodiagnostic severity have yielded inconsistent findings, and the value of CSA for distinguishing consecutive severity categories remains uncertain [10]. We hypothesized that median nerve CSA would be greater in severe CTS than in moderate CTS and would have discriminatory value for differentiating these severity categories. Therefore, this study aimed to evaluate whether median nerve CSA measured ultrasonographically at the pisiform level can distinguish moderate from severe CTS classified according to the Bland electrodiagnostic grading scale. We further investigated the relationship between CSA and electrodiagnostic parameters, including APB needle EMG findings indicative of axonal involvement.

## 2. Methods

### 2.1. Study Design and Patient Selection

This prospective cross-sectional observational study was conducted at the Edirne Sultan 1. Murat State Hospital Electrodiagnostic Laboratory between November 2025 and April 2026. Consecutive patients referred with clinical suspicion of carpal tunnel syndrome (CTS) were prospectively evaluated. Given the exploratory nature of the study, no formal sample size calculation was performed. Following nerve conduction studies (NCS), patients diagnosed with moderate (grade 3), severe (grade 4), or very severe CTS (grade 5) according to the Bland neurophysiological grading scale were eligible for inclusion (Table S1). According to this scale, moderate CTS (grade 3) was defined by a median distal motor latency to the abductor pollicis brevis of >4.5 and <6.5 ms with a preserved index-finger sensory nerve action potential (SNAP); severe CTS (grade 4) by the same distal motor latency range with an absent SNAP; and very severe CTS (grade 5) by a distal motor latency of >6.5 ms [4]. For statistical analysis, patients classified as severe or very severe according to the Bland criteria were combined into a single severe CTS group. To avoid within-subject correlation, only one hand per patient was included. In patients with unilateral symptoms, the symptomatic hand was selected; in those with bilateral symptoms, the hand with the higher Bland grade was included. The study was intentionally restricted to moderate, severe, and very severe CTS because its prespecified objective was to evaluate whether median nerve CSA could distinguish moderate from severe or very severe electrodiagnostic disease and to examine its relationship with motor NCS and APB needle EMG findings; mild CTS was therefore outside the scope of the study.

Patients were excluded if they had a history of previous CTS surgery or local injection, known peripheral neuropathy, diabetes mellitus, thyroid disease, chronic kidney disease, or sonographic evidence of a mass lesion or bifid median nerve. Demographic and clinical data were recorded, including age, symptom duration, Tinel and Phalen sign positivity, and the presence of abductor pollicis brevis (APB) muscle weakness.

Healthy asymptomatic volunteers were recruited as the control group. Controls had no clinical symptoms suggestive of CTS, no known systemic disease associated with neuropathy, no abnormal neurological examination findings, and no abnormal NCS findings. The control group was selected to be comparable to the patient groups with respect to age and sex. All controls underwent both NCS and ultrasound examination. For the control group, the right hand was included in the analysis. The control group was included to provide a reference comparison for median nerve CSA and electrodiagnostic parameters. Written informed consent was obtained from all participants. The study was approved by the Trakya University School of Medicine Ethics Committee (protocol number: 2025/563).

### 2.2. Electrodiagnostic Studies

Electrodiagnostic studies were performed using an electromyography system (Neuropack S1 MEB-9400K, Nihon Kohden Co., Tokyo, Japan) in accordance with standard laboratory protocols. Before NCS, skin temperature was measured over the dorsum of the hand using a non-contact infrared thermometer. If the temperature was below 32 °C, the hand was warmed using a handheld warm-air dryer, and the temperature was reassessed until it reached ≥32 °C. Testing was initiated only after the required skin temperature was reached. The ambient laboratory temperature was kept constant throughout the examinations, although skin temperature was not continuously monitored during testing. NCS were performed by an electrophysiology technician and interpreted by a neurologist (AA), who was blinded to the ultrasound findings.

Median motor responses were recorded from the APB muscle, including distal motor latency and compound muscle action potential (CMAP) amplitude. Median sensory studies were performed antidromically from the second digit, and sensory latency and sensory nerve action potential (SNAP) amplitude were recorded. Ulnar nerve conduction studies were performed in both upper extremities, and lower-extremity nerve conduction studies were added when clinically indicated to exclude polyneuropathy.

Needle electromyography of the APB muscle was performed in all CTS patients using concentric needle electrodes measuring 38 mm in length and 0.45 mm in diameter (26G; Oryx Healthcare, Istanbul, Turkey). The examination was performed by a qualified neurologist (AA) to assess acute denervation, reduced recruitment, and neurogenic motor unit action potentials.

### 2.3. Ultrasound Assessment

Ultrasound examinations were performed on the same day as the electrodiagnostic studies using a Mindray DC-70 ultrasound system (Shenzhen Mindray Bio-Medical Electronics Co., Ltd., Shenzhen, China) equipped with a broadband 3.0–12.0 MHz linear transducer (L12-3E probe). Median nerve cross-sectional area (CSA) measurements were performed by the primary ultrasound examiner, a neurologist (IP) who was blinded to the participants’ clinical and electrodiagnostic findings. The primary examiner and the second neurologist who performed the measurements for the interobserver reliability analysis each had four years of experience in peripheral nerve ultrasonography.

**Figure 1:**
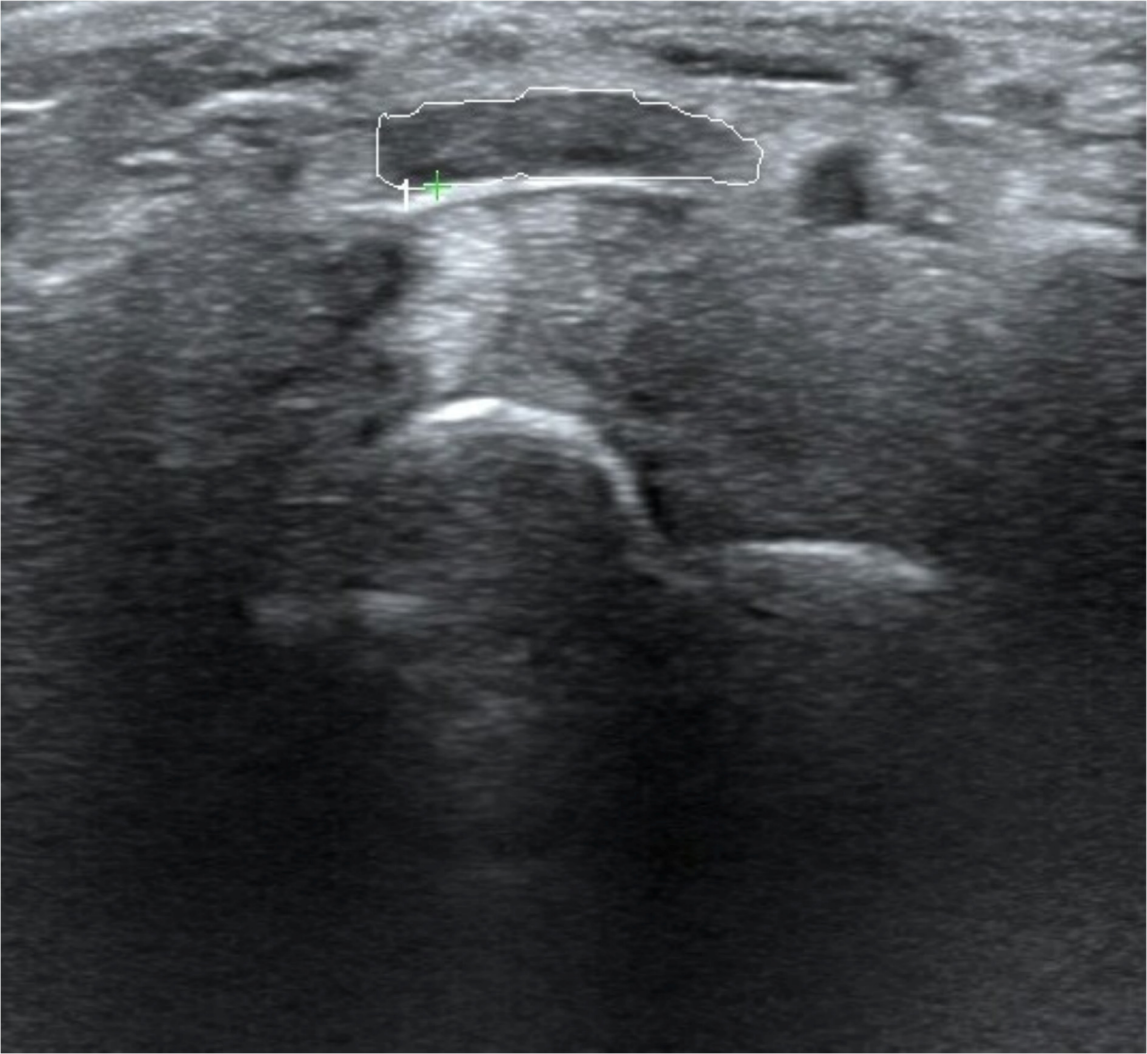
Ultrasonographic measurement of the median nerve cross-sectional area (CSA) at the proximal inlet of the carpal tunnel. The pisiform bone was used as the anatomical landmark, and CSA was obtained by tracing the inner margin of the hyperechoic epineurium to encircle the nerve. The on-screen “+” symbol denotes the trace-measurement cursor, and the numeral “1” identifes the first measurement recorded by the ultrasound system.

Participants were seated with the forearm resting on the examination table in a supinated position, the wrist maintained in a neutral position, and the fingers semi-flexed. Median nerve CSA was measured at the proximal inlet of the carpal tunnel, using the pisiform bone as the anatomical landmark. Three separate measurements were obtained for each included hand. Before each measurement, the transducer was lifted from the skin and repositioned at the same anatomical level, and a new transverse image was acquired, while the hand was maintained in the same standardised position throughout the measurement sequence. Care was taken to keep the transducer perpendicular to the median nerve and to avoid excessive probe pressure. On each frozen image, CSA was measured by manually tracing along the inner border of the hyperechoic epineurium, as illustrated in Figure 1. The mean of the three measurements was used for statistical analysis.

### 2.4. Reliability Assessment

To assess intra-observer reliability, CSA measurements were repeated by the same examiner in a randomly selected subgroup of 10 hands after a one-week interval, with the examiner blinded to the initial measurements. To assess interobserver reliability, CSA measurements were independently repeated in the same subgroup by a second neurologist (AA), who was blinded to the initial CSA measurements obtained by the first examiner but not to the electrodiagnostic findings. Measurements were performed independently during the same session, with repositioning of the transducer before each assessment. Intraobserver reliability was assessed using a single-measurement, absolute-agreement, two-way mixed-effects model, whereas interobserver reliability was assessed using a single-rating, absolute-agreement, two-way random-effects model. Intraclass correlation coefficient (ICC) estimates were reported with their 95% confidence intervals.

### 2.5. Statistical Analysis

Statistical analyses were performed using IBM SPSS Statistics version 23.0 (IBM Corp., Armonk, NY, USA) and GraphPad Prism version 8.4.3 for Windows (GraphPad Software, San Diego, CA, USA). No missing data were present for the variables included in the analyses. The distribution of continuous variables was assessed using the Kolmogorov–Smirnov and Shapiro–Wilk tests. For comparisons between two independent groups, the independent-samples *t*-test or Mann–Whitney U test was used according to data distribution. Normally distributed continuous variables were compared among three or more groups using one-way analysis of variance (ANOVA), whereas non-normally distributed variables were compared using the Kruskal–Wallis H test. Pairwise comparisons were performed using Tukey’s post hoc test after ANOVA and Dunn’s test after the Kruskal–Wallis test, as appropriate. Categorical variables were compared using the chi-square test or Fisher’s exact test, as appropriate.

Pearson or Spearman’s rho correlation coefficients were used to examine associations between continuous variables, depending on data distribution. Exploratory linear regression analysis was performed with median nerve CSA as the dependent variable to determine whether its association with distal motor latency persisted after adjustment for age. Regression model assumptions were assessed by visual inspection of residual plots. Multicollinearity was evaluated using variance inflation factor values.

Receiver operating characteristic (ROC) curve analysis was performed to evaluate the discriminatory performance of median nerve CSA for differentiating severe CTS from moderate CTS. The optimal CSA cut-off value was determined using the Youden index. The area under the curve (AUC), 95% confidence interval, sensitivity, and specificity were calculated. Continuous variables were expressed as mean ± standard deviation or median (minimum–maximum), and categorical variables were expressed as numbers and percentages. A *p*-value of <0.05 was considered statistically significant.

## 3. Results

A total of 72 participants were included and categorised into three groups: moderate CTS (*n* = 34), severe CTS (*n* = 24), and healthy controls (*n* = 14). Female participants accounted for 26 patients (76.5%) in the moderate CTS group, 19 patients (79.2%) in the severe CTS group, and 10 participants (71.4%) in the control group, with no significant difference among groups (*p* = 0.863). The mean age of the overall study population was 50.2 ± 11.4 years, and age did not differ significantly among groups (*p* = 0.078). The clinical and ultrasonographic characteristics of the study groups are presented in Table 1.

During the screening phase before final inclusion, patients with sonographic evidence of a bifid median nerve or a mass lesion were excluded from the analysis. One mass lesion was subsequently confirmed as schwannoma on magnetic resonance imaging (MRI).

Electrodiagnostic characteristics are summarised in Table 2. Distal motor latency, CMAP amplitude, sensory latency, and SNAP amplitude differed significantly among the three groups. In post hoc pairwise comparisons, all NCS parameters except sensory latency differed significantly between the moderate and severe CTS groups. Needle EMG was performed only in participants with CTS. Among needle EMG findings, reduced recruitment was significantly more frequent in the severe CTS group, whereas neurogenic motor unit action potentials were more frequent in the severe CTS group but did not reach statistical significance (*p* = 0.077).

**Figure 2:**
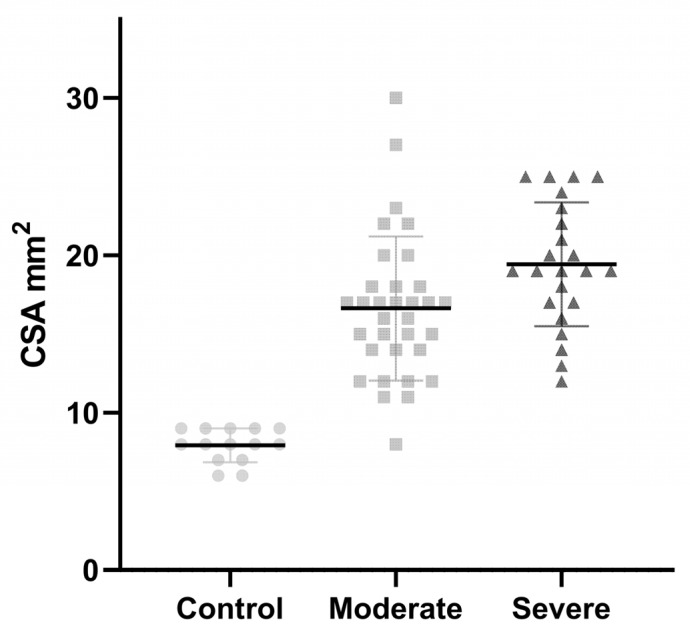
Distribution of median nerve cross-sectional area (CSA) measurements across the control, moderate CTS, and severe CTS groups. Each symbol represents an individual participant. Circles, squares and triangles represent the control, moderate CTS, and severe CTS groups, respectively. Horizontal black lines indicate group means, and error bars represent standard deviations. Although individual CSA measurements overlap between the moderate and severe CTS groups, the mean CSA is higher in the severe CTS group.

Median nerve CSA differed significantly among the three groups (Table 1 and Figure 2), with a progressive increase from healthy controls to moderate and severe CTS groups. Mean CSA was 8.0 ± 1.1 mm^2^ in controls, 16.5 ± 4.6 mm^2^ in the moderate CTS group, and 19.8 ± 3.4 mm^2^ in the severe CTS group (*p* < 0.001). Post hoc pairwise comparisons demonstrated significant differences in CSA between controls and moderate CTS, controls and severe CTS, and moderate and severe CTS groups (all *p* < 0.05). Reliability analysis demonstrated excellent reproducibility of CSA measurements, with intraobserver and interobserver ICC values of 0.95 (95% CI: 0.88–0.98) and 0.91 (95% CI: 0.82–0.96), respectively.

In correlation analyses, median nerve CSA showed moderate positive correlations with distal motor latency and sensory latency (r = 0.624, *p* < 0.001; r = 0.569, *p* < 0.001, respectively). CSA was also inversely correlated with CMAP amplitude and SNAP amplitude (r = −0.589, *p* < 0.001; r = −0.659, *p* < 0.001, respectively). Weak positive associations were observed between CSA and Tinel sign positivity, Phalen sign positivity, and neurogenic motor unit action potentials on needle EMG (Spearman’s rho = 0.325, *p* = 0.006; rho = 0.442, *p* < 0.001; rho = 0.333, *p* = 0.011, respectively).

In the exploratory linear regression model including age and distal motor latency, distal motor latency remained independently associated with median nerve CSA (unstandardised B = 0.873, *p* = 0.002), whereas age was not significantly associated with CSA (unstandardised B = 0.095, *p* = 0.063). No multicollinearity was detected, with variance inflation factor values of 1.00 for both variables (Table 3).

**Figure 3:**
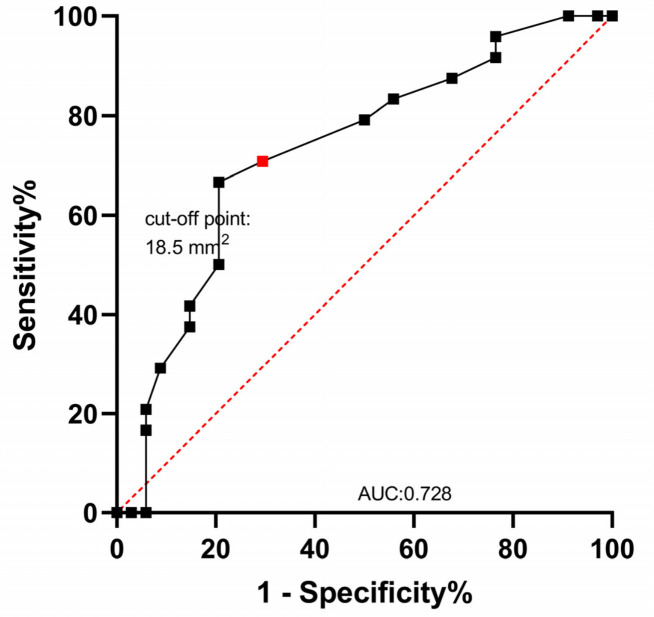
Receiver operating characteristic (ROC) curve of median nerve cross-sectional area for distinguishing severe CTS from moderate CTS. The optimal cut-off value was 18.5 mm^2^, with an area under the curve (AUC) of 0.728, sensitivity of 66.67%, and specificity of 79.41%. The black squares indicate the ROC coordinate points corresponding to the evaluated cut-off values, the red square indicates the selected optimal cut-off value, and the red dashed diagonal line represents the reference line of no discrimination (AUC = 0.5).

ROC curve analysis was performed in 58 patients with moderate or severe CTS and identified an optimal CSA cut-off value of 18.5 mm^2^ for distinguishing severe CTS from moderate CTS. This threshold yielded a sensitivity of 66.67% and a specificity of 79.41%, with an area under the curve of 0.728 (95% CI: 0.594–0.862; *p* = 0.0032; Figure 3).

## 4. Discussion

In this study, median nerve CSA was significantly greater in severe CTS than in moderate CTS and demonstrated moderate discriminatory performance for differentiating these electrodiagnostic severity categories, with an AUC of 0.728 (95% CI: 0.594–0.862). The optimal CSA cut-off value was 18.5 mm^2^, yielding a sensitivity of 66.67% and a specificity of 79.41%. However, the substantial overlap in individual CSA values between the moderate and severe groups and the limited sensitivity indicate that CSA cannot reliably classify severity at the individual-patient level or exclude severe CTS when used alone. Moreover, because this cut-off was derived from a relatively small severe CTS group (*n* = 24) and was not externally validated, it should be regarded as an exploratory, sample-dependent threshold rather than a definitive clinical decision threshold. Accordingly, median nerve CSA should be interpreted as an adjunctive sonographic parameter alongside electrodiagnostic and clinical findings.

Previous studies have primarily evaluated the diagnostic utility of ultrasound for confirming CTS, whereas the role of median nerve CSA in severity stratification remains less clearly defined [12,13]. Recent studies focusing on CTS severity have shown that CSA tends to increase with electrodiagnostic severity and may be particularly useful for identifying severe CTS [14,15]. Rayegani et al. reported progressively higher median nerve CSA values at the carpal tunnel inlet across mild, moderate, and severe CTS groups, with a significant difference between moderate and severe cases [15]. Similarly, Azizi et al. found that median nerve CSA contributed to the classification of CTS severity and identified a CSA threshold of >16 mm^2^ for severe CTS, while also showing that the moderate category remained diagnostically challenging [14]. Our results are consistent with these observations and extend them by specifically addressing the clinically relevant distinction between moderate and severe CTS according to the Bland electrodiagnostic grading scale. The cut-off value of 18.5 mm^2^ identified in our study suggests that greater median nerve enlargement may support the recognition of severe CTS. Nevertheless, the moderate AUC underscores the imperfect overlap between structural nerve enlargement and electrodiagnostic severity, which is expected because CSA and nerve conduction studies assess different dimensions of the disease process.

The correlation analyses further support a structural–functional relationship between median nerve enlargement and electrodiagnostic dysfunction. In our study, CSA was positively correlated with distal motor latency and sensory latency, and inversely correlated with CMAP and SNAP amplitudes. Similar findings were reported by El Habashy et al., who demonstrated positive correlations between wrist CSA and both motor and sensory latencies, as well as negative correlations with motor and sensory amplitudes [16]. These associations suggest that increasing CSA is related not only to conduction delay but also to reduced sensory and motor response amplitudes, which may reflect more advanced median nerve dysfunction and possible axonal involvement. In the exploratory age-adjusted regression model, distal motor latency remained independently associated with CSA, supporting an association between nerve enlargement and motor conduction delay beyond the effect of age. However, the modest explanatory value of the model indicates that CSA accounts for only part of the variability in electrodiagnostic dysfunction. This reinforces the concept that CTS severity is multifactorial and cannot be fully captured by a single structural measurement.

Needle EMG findings provide an additional dimension to severity assessment by reflecting motor axonal involvement, particularly in advanced CTS. In our cohort, reduced recruitment was significantly more frequent in the severe CTS group, whereas neurogenic motor unit action potentials were more frequent in the severe CTS group but did not reach statistical significance (*p* = 0.077). These findings indicate more prominent APB needle EMG abnormalities in patients classified as severe according to the Bland scale. Previous studies evaluating CTS-related axonal loss using APB needle EMG have also reported associations between reduced interference pattern and sonographic parameters, supporting a relationship between motor axonal involvement and median nerve morphology [17,18]. However, acute denervation did not differ significantly between moderate and severe CTS in our study. This is consistent with prior observations suggesting that spontaneous activity may not correlate consistently with CSA [17,18]. Spontaneous activity reflects active or subacute axonal injury and may vary according to the timing of nerve injury, reinnervation, and chronicity of denervation. Therefore, the absence of a significant association between acute denervation and CSA does not exclude motor axonal involvement. The weak positive association between CSA and neurogenic motor unit action potentials in our cohort further suggests a limited but biologically plausible relationship between median nerve enlargement and chronic motor axonal changes.

From a clinical perspective, our findings support the use of ultrasound as a complementary tool rather than a replacement for electrodiagnostic assessment. Although surgical decision-making in typical CTS may sometimes rely primarily on clinical history and examination [19], severity assessment in moderate-to-severe disease often benefits from integrating functional, structural, and clinical information. In this context, CSA values above the identified threshold may support the presence of severe CTS, whereas values below this threshold should not be used to exclude severe disease because of the moderate sensitivity observed in our analysis. Beyond CSA measurement, ultrasound provides direct anatomical assessment of the median nerve and surrounding carpal tunnel structures, including anatomical variants or space-occupying lesions that are not captured by NCS or needle EMG but may influence diagnosis and management [20]. During the screening process for the present study, sonographic assessment identified exclusion findings such as a bifid median nerve and a mass lesion later confirmed as schwannoma on MRI, illustrating the additional anatomical value of ultrasound in the evaluation of patients with suspected CTS.

Although our study focused on CSA, other sonographic parameters may offer complementary value in evaluating CTS. Features such as longitudinal nerve deformity, palmar bowing of the flexor retinaculum, median nerve flattening, intraneural hypervascularity, and reduced echogenicity have been associated with severity [21,22]. Moreover, dynamic ultrasonography can quantify longitudinal and transverse median nerve gliding during wrist or finger movement. Impaired median nerve excursion has been demonstrated in CTS, and its reported association with greater electrodiagnostic severity suggests that mechanical tethering may reflect a functional dimension of the disease not captured by static CSA measurements [23,24]. Because these parameters were not evaluated in the present study, their additional value for distinguishing moderate from severe CTS could not be determined. Future studies incorporating both static morphological markers and dynamic nerve-gliding parameters may provide a more comprehensive sonographic assessment of CTS severity.

This study has several limitations. First, CTS severity was defined according to the Bland electrodiagnostic grading scale; consequently, the ROC analysis evaluated how well CSA discriminated among electrodiagnostically defined severity categories, rather than whether CSA was an independent measure of disease severity. Although CSA was measured independently and is not included in the Bland classification, the absence of an independent clinical, functional, or longitudinal reference outcome precludes determining whether ultrasonography provides incremental clinical or prognostic value beyond electrodiagnostic assessment. Because NCS and ultrasonography assess functional impairment and structural enlargement, respectively, the observed overlap between the moderate and severe groups may reflect both biological variability and differences between the constructs being measured. Second, this was a single-center study with a relatively modest sample size, which may restrict the generalizability of the findings. The control group was also relatively small, although it was selected to be comparable to the patient groups with respect to age and sex. In addition, given the exploratory design, no formal sample size calculation was performed; therefore, the results should be interpreted with caution and validated in larger cohorts. Third, ultrasound assessment was limited to median nerve CSA at the pisiform level, and additional sonographic parameters such as the wrist-to-forearm ratio, flattening ratio, vascularity, and elastography were not evaluated. Incorporating these parameters may improve discrimination between electrodiagnostically defined severity groups in future studies. Fourth, although both intraobserver and interobserver reliability analyses demonstrated excellent agreement and the primary ultrasound examiner was blinded to the clinical and electrodiagnostic findings, the second examiner was not blinded to the electrodiagnostic findings, which may have introduced measurement bias. Fifth, patients with severe and very severe CTS according to the Bland scale were combined into a single severe CTS group for statistical analysis, which may have reduced the ability to explore sonographic differences across the most advanced electrodiagnostic severity categories. Finally, the cross-sectional design precludes conclusions regarding prognosis, surgical outcomes, treatment response, or longitudinal changes in CSA and needle EMG findings. Future studies incorporating independent clinical and functional outcomes and longitudinal follow-up are needed to determine whether CSA adds value beyond electrodiagnostic classification.

## 5. Conclusions

Median nerve CSA measured at the pisiform level may provide useful adjunctive structural information associated with electrodiagnostically defined CTS severity and demonstrated moderate discriminatory performance in distinguishing severe CTS from moderate CTS. Its associations with electrodiagnostic parameters and APB needle EMG abnormalities suggest a structural–functional relationship in CTS. However, CSA should be interpreted in conjunction with clinical findings, NCS, and needle EMG, rather than as a stand-alone substitute for electrodiagnostic assessment. Further studies incorporating independent clinical, functional, and longitudinal outcomes are needed to determine whether CSA provides incremental value beyond electrodiagnostic testing.

## Figures and Tables

**Table 1:** Clinical and Ultrasonographic Characteristics of the Control, Moderate CTS, and Severe CTS Groups.

Variable	Control (*n* = 14)	Moderate CTS (*n* = 34)	Severe CTS (*n* = 24)	*p*-Value
Age, years	46.3 ± 11.6 (28–66)	49.0 ± 11.5 (23–75)	54.2 ± 10.2 (32–70)	0.078
Symptom duration, months	—	9.5 (0.5–60)	12 (1–72)	0.249
Tinel sign, *n* (%)	—	31 (91%)	15 (63%)	0.010
Phalen sign, *n* (%)	—	29 (85%)	17 (71%)	0.156
APB weakness, *n* (%)	—	17 (50%)	21 (88%)	0.005
CSA, mm^2^	8.0 ± 1.1 (6.0–9.0)	16.5 ± 4.6 (8.0–30.0)	19.8 ± 3.4 (12.0–25.0)	<0.001

Data are presented as mean ± standard deviation (minimum–maximum), median (minimum–maximum), or *n* (%), as appropriate. *p*-values for age and CSA were calculated using one-way analysis of variance. Symptom duration was compared between the moderate and severe CTS groups using the Mann–Whitney U test. Categorical variables were compared using the chi-square test. APB, abductor pollicis brevis; CSA, cross-sectional area; CTS, carpal tunnel syndrome.

**Table 2:** Electrodiagnostic and Needle EMG Characteristics of the Study Groups.

Variable	Control (*n* = 14)	Moderate CTS (*n* = 34)	Severe CTS (*n* = 24)	*p*-Value
Distal motor latency, ms	3.0 ± 0.1 ^a^	4.9 ± 1.1 ^b^	7.8 ± 2.1 ^c^	<0.001
CMAP amplitude, mV	17.1 ± 4.6 ^a^	10.5 ± 2.9 ^b^	6.8 ± 3.0 ^c^	<0.001
Sensory latency, ms	2.5 ± 0.2 ^a^	4.1 ± 1.1 ^b^	5.5 ± 2.0 ^b^	<0.001
SNAP amplitude, µV	40.6 ± 10.5 ^a^	15.4 ± 12.1 ^b^	6.1 ± 4.7 ^c^	<0.001
Acute denervation, *n* (%)	—	27 (79%)	20 (83%)	0.491
Reduced recruitment, *n* (%)	—	27 (79%)	24 (100%)	0.018
Neurogenic MUAPs, *n* (%)	—	27 (79%)	23 (96%)	0.077

Data are presented as mean ± standard deviation or *n* (%), as appropriate. Superscript letters indicate post hoc pairwise comparisons; groups sharing the same superscript letter are not significantly different. *p*-values for distal motor latency, CMAP amplitude, and sensory latency were calculated using one-way analysis of variance. SNAP amplitude was compared using the Kruskal–Wallis H test. Needle EMG findings were compared between the moderate and severe CTS groups using the chi-square test. CMAP, compound muscle action potential; CTS, carpal tunnel syndrome; MUAPs, motor unit action potentials; SNAP, sensory nerve action potential.

**Table 3:** Linear Regression Analysis for Median Nerve CSA in Patients with CTS.

Predictor	B	SE	β	t	*p*-Value	95% CI for B	VIF
Constant	7.698	3.007	—	2.560	0.013	1.670 to 13.726	—
Age	0.095	0.050	0.228	1.895	0.063	−0.005 to 0.195	1.001
DML	0.873	0.262	0.401	3.330	0.002	0.347 to 1.399	1.001

Dependent variable: median nerve CSA. B, unstandardised regression coefficient; β, standardised regression coefficient; CI, confidence interval; DML, distal motor latency; SE, standard error; VIF, variance inflation factor.

## Data Availability

The data presented in this study are available upon reasonable request from the corresponding author due to ethical and privacy restrictions concerning patient-level clinical, electrophysiological, and ultrasonographic data.

## Supplementary Materials

The following supporting information can be downloaded at https://www.mdpi.com/article/10.3390/diagnostics16152458/s1, Table S1. Bland Neurophysiological Grading Scale for Carpal Tunnel Syndrome.

## Abbreviations

APB	Abductor pollicis brevis
AUC	Area under the curve
CTS	Carpal tunnel syndrome
CMAP	Compound muscle action potential
CSA	Cross-sectional area
EMG	Electromyography
ICC	Intraclass correlation coefficient
MRI	Magnetic resonance imaging
NCS	Nerve conduction studies
ROC	Receiver operating characteristic
SNAP	Sensory nerve action potential
US	Ultrasound
