# Supplementary material for: Ultrasound-Based Severity Stratification in Carpal Tunnel Syndrome: Median Nerve Cross-Sectional Area for Distinguishing Severe from Moderate Disease
Source: Diagnostics (Basel). 2026 Aug 4;16(15):2458. doi: 10.3390/diagnostics16152458 (PMC13465334; doi:10.3390/diagnostics16152458)
Supplement: Supplementary file 1 [file diagnostics-16-02458-s001.zip › diagnostics-4459485-supplementary.pdf]

Supplementary Table S1. Bland Neurophysiological Grading Scale for Carpal Tunnel Syndrome

Grade	Severity category	Nerve conduction findings
0	Normal	Normal nerve conduction findings
1	Very mild CTS	CTS demonstrable only with the most sensitive comparative or segmental tests
2	Mild CTS	Slowed sensory nerve conduction velocity on finger–wrist measurement, with normal distal motor latency
3	Moderate CTS	Sensory potential preserved with motor slowing; DML to APB < 6.5 ms
4	Severe CTS	Sensory potentials absent, but motor responses preserved; DML to APB < 6.5 ms
5	Very severe CTS	Sensory potentials absent and DML to APB > 6.5 ms
6	Extremely severe CTS	Sensory and motor potentials effectively unrecordable

CTS, carpal tunnel syndrome; APB, abductor pollicis brevis; DML, distal motor latency; ms, milliseconds.
